# ADAMTS proteoglycanases in the physiological and pathological central nervous system

**DOI:** 10.1186/1742-2094-10-133

**Published:** 2013-10-31

**Authors:** Sighild Lemarchant, Mathilde Pruvost, Joan Montaner, Evelyne Emery, Denis Vivien, Katja Kanninen, Jari Koistinaho

**Affiliations:** 1 Department of Neurobiology, A. I. Virtanen Institute for Molecular Sciences, Biocenter Kuopio, University of Eastern Finland, P.O. Box 1627, 70211 Kuopio, Finland; 2 INSERM, INSERM UMR-S 919, “Serine Proteases and Pathophysiology of the neurovascular Unit”, University of Caen Basse-Normandie, GIP Cyceron, Bd H. Becquerel, BP 5229, 14074 Caen Cedex, France; 3 Neurovascular Research Laboratory, Vall d’Hebron Research Institute, Universitat Autonoma de Barcelona, Barcelona, Spain; 4 Department of Neurosurgery, Caen University Hospital, Avenue de la Côte de Nacre, 14000 Caen, France; 5 Department of Oncology, Kuopio University Hospital, P.O. Box 1777, 70211 Kuopio, Finland

**Keywords:** A disintegrin and metalloproteinase with thrombospondin motifs, Proteoglycanases, Stroke, Spinal cord injury, Chondroitin sulfate proteoglycan, Synaptic plasticity, Inflammation, Angiogenesis, Neurorepair

## Abstract

ADAMTS-1, -4, -5 and -9 belong to ‘a disintegrin and metalloproteinase with thrombospondin motifs (ADAMTS)’ family and more precisely to the proteoglycanases subgroup based on their common ability to degrade chondroitin sulfate proteoglycans. They have been extensively investigated for their involvement in inflammation-induced osteoarthritis, and a growing body of evidence indicates that they may be of key importance in the physiological and pathological central nervous system (CNS). In this review, we discuss the deregulated expression of ADAMTS proteoglycanases during acute CNS injuries, such as stroke and spinal cord injury. Then, we provide new insights on ADAMTS proteoglycanases mediating synaptic plasticity, neurorepair, angiogenesis and inflammation mechanisms. Altogether, this review allows us to propose that ADAMTS proteoglycanases may be original therapeutic targets for CNS injuries.

## Introduction

The ADAMTS proteases belong to ‘a disintegrin and metalloproteinase with thrombospondin motifs’ family, composed of 19 members. They are multi-domain proteins synthesized as pre-pro-enzymes containing from the N- to the C-terminal end: a peptide signal, a pro-domain, a zinc binding metalloproteinase domain, a disintegrin-like domain, a thrombospondin domain, a cysteine-rich domain, a spacer domain and finally a variable number of thrombospondin motifs in the C-terminal end [1,2]. Pre-pro-ADAMTS proteases can be cleaved by furin or furin-like proteases at the N- and C-terminal positions triggering, respectively, their activity and their future location within the extracellular matrix (ECM) via the removal of the pro-domain, and their substrate specificity [3-11]. When secreted, ADAMTS proteases are capable of binding ECM components via their thrombospondin motifs [3], which can be then cleaved by the metalloproteinase domain. ADAMTS proteases are classified in three subfamilies based on their preference to cleave specific ECM macromolecules as follows [12-14]: proteoglycans (ADAMTS-1, -4, -5, -8, -9, -15 and -20), pro-collagens (ADAMTS-2, -3 and -14) or the von Willebrand factor (ADAMTS-13). This review will focus on the ADAMTS proteoglycanases present in the central nervous system (CNS), with special emphasis on ADAMTS-1, -4, -5 and -9, which are key enzymes in the degradation of the aggregating chondroitin sulfate proteoglycans (CSPGs) [14].

CSPGs are a family of ECM macromolecules characterized by a core protein and a variable number of glycosaminoglycan chains. The major CSPGs found in the CNS are lecticans (for example, aggrecan, brevican, neurocan and versican), phosphocan and type 2 neuroglycan. They are concentrated into perineuronal nets (PNNs) that enwrap a subset of neurons and control the path finding and guidance of axons during CNS development. After CNS injury, CSPGs are rapidly upregulated within the glial scar and exert both beneficial and deleterious effects. For instance, their contribution to the establishment of a dense glial scar initially constitutes a protective barrier to limit the propagation of damage, but also represents a harmful barrier to subsequent neurorepair and neuroplasticity [15].

While ADAMTS proteoglycanases have been extensively described for their deleterious effect in osteoarthritis, rheumatoid arthritis and vertebral disc degeneration, it is only recently that their role in the CNS has been discussed. Therefore, in this review we will provide up-to-date information about the increasing evidence for the involvement of ADAMTS proteoglycanases in physiological conditions and CNS pathological disease states including ischemic stroke and spinal cord injury (SCI).

### ADAMTS proteoglycanases in the physiological central nervous system

ADAMTS proteoglycanases are present in several CNS structures, including the cortex, the hippocampus, the striatum and the spinal cord [16-21]. While it is clear that astrocytes express ADAMTS proteoglycanases *in vitro* and *in vivo*[22-25], their presence in neurons under physiological conditions is controversial. ADAMTS-4 was identified in dentate granule neurons and pyramidal cells by *in situ* hybridization in rat brains [16]. Similarly, the expression of ADAMTS-4 was described in cortical neurons *in vitro*[25], but a more recent investigation failed to detect ADAMTS-4 in cerebellar granule neurons *in vitro*[24]. The presence of ADAMTS-4 has also been reported in cortical microglia *in vitro*[24,25] (Table 1).

**Table 1:** Cellular expression of ADAMTS proteoglycanases in the physiological and pathological central nervous system

**Cell type**			**ADAMTS-1**	**ADAMTS-4**	**ADAMTS-5**	**ADAMTS-9**
**Astrocytes**	*In vitro:*	Primary cerebral cultures (mouse) [34]	Yes (mRNA)	Yes (mRNA)	Yes (mRNA)	Yes (mRNA)
		Primary cortical cultures (rat) [24,25]		Yes (mRNA/WB)		
		Primary cerebral cultures (human) [23]:	Yes (mRNA/WB/ICC)	Yes (mRNA/WB/ICC)	Yes (mRNA/WB/ICC)	
		- TNF-α treatment	↑ mRNA	↑ mRNA	stable mRNA	
			stable protein	↑ protein	stable protein	
		- IL1-β treatment	stable mRNA	stable mRNA	stable mRNA	
	*In vivo:*	Brain white matter (human) [22]	Yes (IHC)	Yes (IHC)	Yes (IHC)	
		Spinal cord white matter (rat) [21]	Yes (IHC)	Yes (IHC)	Yes (IHC)	
		Injured brain, tMCAO (mouse) [33]		Yes (mRNA)	Yes (mRNA)	
		Injured cerebral cortex, tMCAO (rat) [32]				Yes (mRNA)
		Injured spinal cord, SCI by contusion (mouse) [34]	Yes (IHC)		Yes (IHC)	Yes (IHC)
**Neurons**	*In vitro:*	Primary cortical cultures (rat) [25]		Yes (WB)		
		Primary cerebellar granule neurons cultures (rat) [24]		No (mRNA)		
	*In vivo:*	Hippocampus, dentate granule neurons and pyramidal neurons (rat) [16]:				
		- Physiological conditions	No (mRNA)	Yes (mRNA)		
		- Kainate-induced CNS excitoxicity	Yes (mRNA)	Yes (mRNA)		
		Injured cerebral cortex, tMCAO (rat) [32]				Yes (mRNA)
		Injured spinal cord, SCI by contusion (mouse) [34]				No (IHC)
**Microglia**	*In vitro:*	Primary cerebral/cortical cultures (rat) [24,25]		Yes (mRNA/WB)		
**Monocytes /**	*In vitro:*	THP-1 monocyte cell line (human) [36,37]	Yes (mRNA)	Yes (mRNA/WB)	Yes (mRNA)	Yes (mRNA)
**Macrophages**	*In vitro:*	THP-1-derived macrophages (human) [36,37]:	Yes (mRNA)	Yes (mRNA/WB)	Yes (mRNA)	Yes (mRNA)
		- TGF-β treatment [36,38]	↑ mRNA	↑ mRNA/↓ mRNA	↑ mRNA	↑ mRNA
		- IFN-ɣ treatment [36,37]	↓ mRNA	stable/↑ mRNA	stable mRNA	↑ mRNA
		- TNF-α treatment [37]		↑ mRNA		slight ↑ mRNA
		- IL1-β treatment [37]	slight ↑ mRNA	stable mRNA		slight ↑ mRNA

This table depicts the various cell types expressing the major ADAMTS proteoglycanases (ADAMTS-1, -4, -5 and -9) *in vitro* and/or *in vivo* under physiological and/or pathological CNS conditions. It also indicates if ADAMTS proteoglycanases were detected at the mRNA level or at the protein level (WB, ICC, IHC). Numbers indicate the concerned references. *ADAMTS*, a disintegrin and metalloproteinase with thrombospondin motifs; *CNS*, central nervous system; *ICC/IHC*, Immunocyto/histochemistry; *IFN-ɣ*, ɣ-interferon; *IL-1β*, interleukin-1β; *SCI*, spinal cord injury; *TGF-β*, transforming growth factor-β; *tMCAO*, transient middle cerebral artery occlusion; *TNF-α*, tumor necrosis factor-α; *WB*, western blot.

In the physiological CNS, evidence exists for a role of ADAMTS proteoglycanases in neural plasticity *in vitro* and *in vivo*. Yuan and collaborators were the first to discover evidence of the presence of ADAMTS-1, -4 and ADAMTS-cleaved brevican fragments in the physiological CNS in rats, particularly in plastic regions such as the hippocampus, suggesting the involvement of the ADAMTS proteoglycanases in the malleability of PNNs-containing brevican [16]. Hamel and collaborators (2008) discovered that a recombinant active ADAMTS-4 promoted neurite growth of cortical neurons *in vitro* i) by degrading CSPGs via its proteolytic activity, and ii) by activating the MAP/ERK (mitogen activated protein/extracellular signal-regulated kinase) signaling pathway, presumably due to the activation of tyrosin kinase receptors by the thrombospondin domain of ADAMTS-4 [26]. However, the concept of CSPGs/ADAMTS proteoglycanases working in concert to regulate plasticity has been explored only recently *in vivo*[19]. Interestingly, the expression of synaptic proteins, such as synaptosomal-associated protein 25 (SNAP-25), postsynaptic density protein 95 (PSD-95) and synaptophysin, was decreased in the developing frontal cortex of ADAMTS-1 deficient female mice, but not in male mice, suggesting a gender-specific involvement of ADAMTS-1 in synaptic plasticity. However, the decline in expression of synaptic proteins was not accompanied by any modifications of CSPGs present in PNNs, or by deficits of learning and memory [19]. Recently, Krstic and collaborators (2012) proposed ADAMTS-4 and ADAMTS-5 as proteases capable of cleaving Reelin, an extracellular molecule also involved in neurodevelopment and in synaptic plasticity induced learning and memory processes [20]. Interestingly, ADAMTS-induced cleavage of Reelin is thought to partly promote its aggregation during aging, and to participate in the well-known synaptic plasticity defects in elderly CNS tissues [20].

To summarize, ADAMTS proteoglycanases in the physiological CNS are synthesized mainly by astrocytes and expressed in several CNS structures. Interestingly, increasing evidence suggests that they may play critical roles in the control of synaptic plasticity during development and aging via both proteolytic-dependent and -independent mechanisms.

### ADAMTS proteoglycanases in the pathological central nervous system

Proteolysis of the ECM can be both beneficial and harmful in several pathological states of the CNS, including ischemic stroke and SCI [27]. A tight control of the local environment is crucial to ensure a moderate remodeling of the ECM in order to promote neuronal plasticity and survival, or vascular remodeling, after acute CNS injuries. While the expression and associated beneficial or deleterious effects of matrix metalloproteinases (MMPs) have been extensively reported in several CNS diseases, recent publications strongly suggest that the ADAMTS proteoglycanases may also be important in ECM proteolysis in CNS injuries.

### ADAMTS proteoglycanases: cytokine- and cell-specific inducible proteases

Several cytokines, including IL-1β (interleukin-1β), IL-6, IFN-ɣ (ɣ-interferon), TGF-β (transforming growth factor-β) and TNF-α (tumor necrosis factor-α), have previously been described to regulate the expression of ADAMTS proteoglycanases in non-CNS cell types [28-31]. The cytokine-rich environment following CNS injuries is therefore likely to induce the expression of a complex pattern of ADAMTS proteoglycanases in a cell- and cytokine-dependent manner. An increased synthesis of ADAMTS-4, -5 and -9 by astrocytes was reported after transient middle cerebral artery occlusion (tMCAO) [32,33]. Similarly, injured neurons were described to synthesize ADAMTS-9 after tMCAO [32], but not after contusion-induced SCI [34]. Interestingly, ADAMTS-1 was also specifically upregulated in cerebral motor neurons after peripheral nerve injury [35]. Few cytokines have been already proposed to regulate ADAMTS proteoglycanases expression in the CNS (Table 1). IL-1α in combination with IL-1 receptor type 1 promotes ADAMTS-1 transcription in a N1E-115 neuroblast cell line *in vitro* and in motoneurons after nerve injury *in vivo*[35]. ADAMTS-4 mRNA and protein expressions were increased by TNF-α in human astrocyte cultures after 24 hours of treatment, while only upregulation of ADAMTS-1 mRNA or ADAMTS-5 protein levels were reported. Under similar conditions, IL-1β did not regulate the transcription of ADAMTS proteoglycanases, although it seems that there is a trend for an increased mRNA expression of ADAMTS-4 [23]. Beyond astrocytes, microglia and neurons, macrophages that infiltrate brain or spinal cord after injury may also be an important source of ADAMTS-1, -4 and -5 [36,37]. Interestingly, while TNF-α and IFN-ɣ increase the expression of ADAMTS-4 in macrophages induced by differentiation of human monocytic cell line THP1 [37], TGF-β negatively regulates ADAMTS-4 expression through the MAPK and the Smad-2 and -3 signaling pathways [36]. In similar conditions, it was also described that TGF-β can increase the synthesis of ADAMTS-4 [38].

Accordingly, modification of ADAMTS proteoglycanases expression has been reported after CNS injuries including stroke and SCI (Table 2). Yuan and collaborators were first to discover evidence of the increase of ADAMTS-1, -4 and ADAMTS-cleaved brevican fragments after intraperitoneal injection of kainate-induced CNS excitoxicity, in the cortex and in the hippocampus, by pyramidal neurons and dentate granule neurons [16]. After tMCAO, the expression of ADAMTS-1, -4, and -9 is upregulated [23,32]. Surprisingly, whereas mRNA changes were observed in the ipsilateral hemisphere acutely at 6 and 24 hours after stroke onset, the upregulation of the protein levels of ADAMTS-1 and -4 was only observed 5 days after injury. Because TNF-α was shown to promote ADAMTS-1 and -4 mRNA levels as well as ADAMTS-4 and -5 protein levels in human astrocyte cultures, it was hypothesized that the increase of TNF-α expression in the acute phase of stroke may be responsible for the upregulation of ADAMTS-1 and -4 by astrocytes [23]. Surprisingly, it is only recently that the upregulation of ADAMTS-4 and -5 mRNA levels were detected in astrocytes after tMCAO in mice 24 hours after stroke onset [33]. The expression of ADAMTS-9 was increased in the acute phase of stroke within 24 hours post occlusion at both the mRNA and protein levels, but rather than astrocytes, neurons were predominantly affected in both contralateral and ipsilateral hemispheres [32]. Similarly, upregulation of ADAMTS-1, -5 and -9 mRNA levels were reported in the acute phase of contusion-induced SCI in mice, whereas no changes to ADAMTS-4 expression were observed [34]. Tauchi and colleagues (2012) detected a slight increase of ADAMTS-4 protein levels associated with a significant increase in ADAMTS-4 enzymatic activity in spinal cord lysates from the lesion site one week after contusion-induced SCI in rats [24]. Demircan and colleagues (2013) also described the astrocytes as being a main source of ADAMTS-1, -5 and -9 in the spinal cord after SCI. However, they did not observe the presence of ADAMTS-9 in neurons after SCI [34].

**Table 2:** Deregulated expression of ADAMTS proteoglycanases after central nervous system injuries

	**STROKE**		**SCI**	
	**tMCAO model**		**Contusion model**	
	**Early stage**^**a**^	**Late stage**^**b**^	**Early stage**^**c**^	**Late stage**^**d**^
**ADAMTS-1**				
*mRNA level*	↑ (rat) [23]	stable (rat) [23]	↑ (mouse) [34]	
*Protein level*	stable (rat) [23]	↑ (rat) [23]		
**ADAMTS-4**				
*mRNA level*	↑ (rat/mouse) [23,33]	stable (rat) [23]	stable (mouse) [34]	
*Protein level*	stable (rat) [23]	↑ (rat) [23]		slight ↑ (rat) [24]
*Activity*				↑ (rat) [24]
**ADAMTS-5**				
*mRNA level*	↑ (mouse) [33]	slight ↑ (rat) [23]	↑ (mouse) [34]	
*Protein level*				
**ADAMTS-9**				
*mRNA level*	↑ (rat) [32]		↑ (mouse) [34]	
*Protein level*	↑ (rat) [32]	not detected (rat) [32]		

This table depicts the expression and/or activity of the major ADAMTS proteoglycanases (ADAMTS-1, -4, -5 and -9) in the time course of CNS injuries, including ischemic stroke (tMCAO model) and spinal cord injury (contusion model). ^a^6 to 24 h, ^b^5 to 7d after stroke onset; ^c^3 to 24 h, ^d^7d after SCI onset. Numbers indicate the concerned references. *ADAMTS*, a disintegrin and metalloproteinase with thrombospondin motifs; *CNS*, central nervous system; *SCI*, spinal cord injury; *tMCAO*, transient middle cerebral artery occlusion.

**Figure 1:**
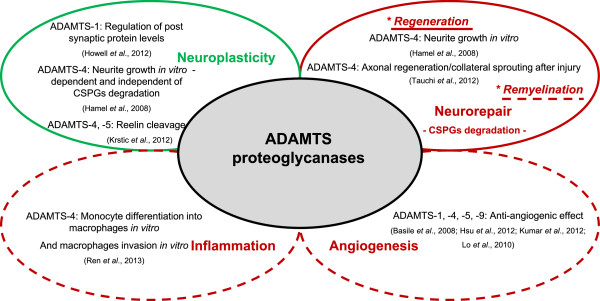
**Roles of ADAMTS proteoglycanases in the physiological and pathological central nervous system.** Schematic representation of described (filled lines)/hypothetical (dotted lines) roles of ADAMTS proteoglycanases in the physiological (green) and pathological (red) CNS, with corresponding major references listed below. This schema also illustrates that ADAMTS proteoglycanases can achieve several functions in the physiological and pathological CNS via the cleavage of their substrates, so far CSPGs or Reelin, but also independently of their proteolytic activity. ADAMTS, a disintegrin and metalloproteinase with thrombospondin motifs; CNS, central nervous system; CSPGs, chondroitin sulfate proteoglycans.

To summarize, CNS injuries including ischemic stroke or SCI lead to the upregulation of different combinations of ADAMTS proteoglycanases, respectively ADAMTS-1, -4, -9 and ADAMTS-1, -5, -9 (Table 2). A cytokine regulation and/or cell-specific expression of ADAMTS proteoglycanases expression strongly suggest that each of them may be associated to a specific local turnover of CSPGs, assuming that they have non-redundant and cooperative functions in the CNS. Several clues indicate that ADAMTS proteoglycanases may have both beneficial and deleterious effects after CNS injuries as described hereafter and summarized in Figure 1.

### ADAMTS proteoglycanases: inflammatory and anti-angiogenic proteases in the central nervous system?

#### ADAMTS proteoglycanases and macrophage infiltration

A growing body of evidence suggests that ADAMTS proteoglycanases may be involved in the neuroinflammatory response after CNS injury by promoting the infiltration of macrophages into the CNS:

1. ADAMTS-4 is increased during the differentiation of human monocytes into macrophages *in vitro*[37] and is required for macrophages invasion *in vitro*[38].

2. Versican is the primary CSPG present in the vasculature and is a potent substrate for ADAMTS proteoglycanases [39], which begs the question of whether upregulation of the ADAMTS proteoglycanases at the neurovascular unit may participate in the leakage of the blood brain/spinal cord barrier after ischemic stroke or SCI. It would be consistent with local degradation of versican by ADAMTS proteoglycanases synthesized by endothelial cells [40-42] and/or monocytes/macrophages [36].

Altogether, it can be hypothesized that ADAMTS proteoglycanases, particularly ADAMTS-4, may be a key player of the macrophage infiltration into the CNS after injury either directly or indirectly by promoting the leakage of the blood brain/spinal cord barrier.

#### ADAMTS proteoglycanases and angiogenesis

The involvement of ADAMTS proteases has been largely investigated in angiogenesis mechanisms occurring in cancer, yet similar mechanisms may also occur in the CNS. Both proteinase-dependent and independent anti-angiogenic functions for most of the ADAMTS proteases, including proteoglycanases, have been described [42-45]. For instance, it has been proposed that ADAMTS proteoglycanases can negatively regulate angiogenesis via the sequestration of the most potent pro-angiogenic factor, VEGF (vascular endothelial growth factor) or via the release of anti-angiogenic fragments derived from thrombospondin type 1 and 2 motifs. Besides its pro-angiogenic effect, VEGF has also been shown to promote vascular permeability, neuroinflammation, neuritic growth and neuroprotection particularly after ischemic stroke or SCI [46]. Therefore, it is possible to hypothesize that the sequestration of VEGF by endogenous ADAMTS proteoglycanases may have both protective and harmful effects in the CNS. To conclude, the effect of ADAMTS proteoglycanases in angiogenesis processes after acute CNS injuries remains largely unknown and deserves further investigation.

### ADAMTS proteoglycanases and neurorepair

Neurorepair including neuroregeneration and remyelination is compromised in the chronic phase of stroke or SCI partly because of an overexpression of CSPGs [47,48]. In parallel with the upregulation of CSPGs within the glial scar, ischemic stroke is also associated with degradation of PNNs containing CSPGs at the lesion site, but also in the peri-ischemic area and the contralateral hemisphere after permanent MCAO. Although the degradation of the PNNs occurred predominantly in the lesion core and was associated with the invasion of monocytes/macrophages, the transient reduction of neurons containing PNNs in the peri-ischemic area or the contralateral hemisphere is more likely associated with an attempt at neurorepair [49]. It is tempting to hypothesize that after CNS injuries, the loss of PNNs may be caused by increased local secretion of neuronal ADAMTS proteoglycanases (as described for ADAMTS-1 and ADAMTS-9) triggering adverse effects: neuroinflammation in the lesion core and an attempt at neuroplasticity in surrounding tissues. However, these attempts at axonal regeneration/collateral sprouting are too transient and/or unsuccessful to efficiently penetrate the repellant CSPGs-rich glial scar and to improve long term functional recovery.

The inhibition of CSPGs can be relieved by the bacterial enzyme chondroitinase ABC which removes the chondroitin sulfate chains from the core proteins, thus promoting axonal regeneration/collateral sprouting of a wide variety of neuron tracts and functional recovery after stroke or SCI [49-57]. However, the core proteins remain intact and can still inhibit neuroregeneration/remyelination. ADAMTS proteoglycanases are physiological enzymes capable of achieving the complete degradation of CSPGs [1,2]. Surprisingly, evidence that ADAMTS proteoglycanases may improve neurite growth and axonal regeneration/collateral sprouting has recently emerged:

1. Hamel and collaborators (2008) reported that a recombinant active ADAMTS-4 can promote neurite growth of cortical neurons *in vitro* dependently or independently of its proteolytic activity as described above [26].

2. Cua and collaborators (2013) reported that ADAMTS-4 was more efficient at degrading CSPGs and inducing subsequent neurite growth *in vitro* than chondroitinase ABC or MMPs [58].

3. The treatment of mice submitted to contusion-induced SCI with recombinant ADAMTS-4 improves axonal regeneration/collateral sprouting of serotoninergic fibers and subsequent functional recovery via the degradation of neurocan [24].

In addition to the inhibition of axonal regeneration/collateral sprouting in CNS injuries, CSPGs are also strongly upregulated within the white matter where they contribute to the inhibition of remyelination of injured axons. Interestingly, chondroitinase ABC can prevent CSPGs-inhibition of remyelination *in vitro* and *in vivo* after contusion-induced SCI in rats and in a lysolecithin-induced demyelination model in mice [47,48,59]. Moreover, olfactory ensheathing cell-based therapies promote remyelination after acute injuries [60] and these cells were recently reported to express ADAMTS-4 [61,62]. This raises the question of whether ADAMTS-4 or any ADAMTS proteoglycanases could improve remyelination of injured axons more efficiently than chondroitinase ABC does.

To conclude, the overexpression of ADAMTS proteoglycanases induced by acute CNS injuries such as stroke or SCI seems harmful in the acute phase through enhancing neuroinflammation, and beneficial (or at least safe) in later phases to initiate neurorepair (Figure 1). A combination approach may represent an attractive therapeutic opportunity: first, the administration of an inhibitor of ADAMTS proteoglycanases activity, such as TIMP-3 (tissue inhibitor of metalloproteinases-3) [63], or an inhibitor of their synthesis, such as the anti-inflammatory compound WIN-34B [29], in the acute phase of CNS injuries to overcome ADAMTS-induced macrophage infiltration; then, the administration of active ADAMTS proteoglycanases in the chronic phase of CNS injuries to enhance neuroregeneration/neuroplasticity/remyelination, by using already commercially available recombinant proteins, or by using lentiviral gene therapy approach in cell transplantation-based therapies. However, the administration of any therapeutic molecule to treat CNS disorders is challenging; in addition to finding the most appropriate timing, their passage across the blood brain/spinal cord barrier can also be problematic [64]. However, nasal delivery of therapeutic molecules to the brain allows them to bypass the barrier and represents a safe and convenient system for pre-clinical and clinical studies [65].

## Conclusions

Exciting evidence for the involvement of ADAMTS proteoglycanases in the CNS in mechanisms governing synaptic plasticity during development and aging has been proposed quite recently and has emphasized the proteolytic-dependent and -independent actions of these ADAMTS proteoglycanases. However, more research is required to determine whether ADAMTS proteoglycanases have redundant spatiotemporal expressions and functions in the physiological and also pathological CNS. After CNS injuries such as stroke and SCI, it seems obvious that ADAMTS proteoglycanases may be detrimental in the acute phase of injury while they may have a beneficial role later on as summarized in Figure 1. Even though their roles in angiogenesis and macrophage infiltration within the injured CNS have not been addressed yet, the early inhibition of ADAMTS proteoglycanases synthesis/activity after brain or spinal cord injuries may represent a rational therapeutic approach to limit the invasion of macrophages and/or to promote angiogenesis. This review clearly supports the postulate that the ADAMTS proteoglycanases, in particular ADAMTS-4, should be considered as key proteases to promote neurorepair after CNS injuries.

## Abbreviations

ADAMTS: A disintegrin and metalloproteinase with thrombospondin motifs; CNS: Central nervous system; CSPG: Chondroitin sulfate proteoglycan; ECM: Extracellular matrix; ERK: Extracellular signal-regulated kinase; IFN-ɣ: ɣ-Interferon; IL-1: Interleukin-1; MAP: Mitogen activated protein; MAPK: Mitogen activated protein kinase; MMP: Matrix metalloproteinase; NF-κB: Nuclear factor kappa-light-chain-enhancer of activated B cells; PNN: Perineuronal net; PSD-95: Postsynaptic density protein 95; SCI: Spinal cord injury; SNAP-25: Synaptosomal-associated protein 25; TGF-β: Transforming growth factor-β; TIMP-3: Tissue inhibitor of metalloproteinases-3; tMCAO: Transient middle cerebral artery occlusion; TNF-α: Tumor necrosis factor-α; VEGF: Vascular endothelial growth factor.

## Competing interests

The authors declare that they have no competing interests.

## Authors’ contributions

SL planned and wrote the review. All authors gave critical comments on the draft of the manuscript based on their expertise on pre-clinical (SL, MP, DV, KK, JK) and/or clinical studies (JM, EE) on stroke and/or spinal cord injuries. SL and MP prepared the tables/figures. KK helped in editing the manuscript. All authors read and approved the final version of the manuscript.
